# Optimizing percutaneous pulmonary valve implantation with patient-specific 3D-printed pulmonary artery models and hemodynamic assessment

**DOI:** 10.3389/fcvm.2023.1331206

**Published:** 2024-01-08

**Authors:** Ender Odemis, İbrahim Basar AKA, Mhd Homam Alhaj Ali, Terman Gumus, Kerem Pekkan

**Affiliations:** ^1^ Congenital Heart Disease Research Laboratory, Kuttam, Koç University Hospital, Istanbul, Türkiye; ^2^ Department of Pediatric Cardiology, Faculty of Medicine, Koç University, Istanbul, Türkiye; ^3^ Department of Mechatronics Engineering, Faculty of Engineering and Natural Sciences, İstanbul Bilgi University, Istanbul, Türkiye; ^4^ Biomedical Engineering, Faculty of Engineering and Natural Sciences, İstanbul Medipol University, Istanbul, Türkiye; ^5^ Department of Radiology, Faculty of Medicine, Koç University, Istanbul, Türkiye; ^6^ Department of Mechanical Engineering, Faculty of Engineering, Koç University, Istanbul, Türkiye

**Keywords:** percutaneous pulmonary valve implantation, tetralogy of fallot, 3D models, *in vitro* hemodynamic, ViVitro percutaneous pulmonary valve implantation, hemodynamic, ViVitro, Pulsta

## Abstract

**Background:**

Percutaneous pulmonary valve implantation (PPVI) has emerged as a less invasive alternative for treating severe pulmonary regurgitation after tetralogy of Fallot (TOF) repair in patients with a native right ventricular outflow tract (RVOT). However, the success of PPVI depends on precise patient-specific valve sizing, the avoidance of oversizing complications, and optimal valve performance. In recent years, innovative adaptations of commercially available cardiovascular mock loops have been used to test conduits in the pulmonary position. These models are instrumental in facilitating accurate pulmonic valve sizing, mitigating the risk of oversizing, and providing insight into the valve performance before implantation. This study explored the utilization of custom-modified mock loops to implant patient-specific 3D-printed pulmonary artery geometries, thereby advancing PPVI planning and execution.

**Material and Methods:**

Patient-specific 3D-printed pulmonary artery geometries of five patients who underwent PPVI using Pulsta transcatheter heart valve (THV) ® were tested in a modified ViVitro pulse duplicator system®. Various valve sizes were subjected to 10 cycles of testing at different cardiac output levels. The transpulmonary systolic and regurgitation fractions of the valves were also recorded and compared.

**Results:**

A total of 39 experiments were conducted using five different patient geometries and several different valve sizes (26, 28, 30, and 32 mm) at 3, 4, and 5 L/min cardiac output at heart rates of 70 beats per minute (bpm) and 60/40 systolic/diastolic ratios. The pressure gradients and regurgitation fractions of the tested valve sizes in the models were found to be similar to the pressure gradients and regurgitation fractions of valves used in real procedures. However, in two patients, different valve sizes showed better hemodynamic values than the actual implanted valves.

**Discussion:**

The use of 3D printing technology, electromagnetic flow meters, and the custom-modified ViVitro pulse duplicator system® in conjunction with patient-specific pulmonary artery models has enabled a comprehensive assessment of percutaneous pulmonic valve implantation performance. This approach allows for accurate valve sizing, minimization of oversizing risks, and valuable insights into hemodynamic behavior before implantation. The data obtained from this experimental setup will contribute to advancing PPVI procedures and offer potential benefits in improving patient outcomes and safety.

## Introduction

Although the early survival rate following tetralogy of Fallot (TOF) repair is excellent, long-term survival beyond the second decade is lower than that of the healthy population (1). Right ventricular outflow (RVOT) patch is a well-defined risk factor for sudden death related to chronic severe pulmonary (PR) regurgitation due to right ventricular dilatation, ventricular arrhythmia, and heart failure (2). The sudden cardiac death ratio has been reported as 0.02% annually, which is considerably higher in patients with transannular patches (3, 4). Therefore, pulmonary valve implantation (PVI) is a Class I priority in symptomatic patients. Asymptomatic patients also have indications for PVI in the presence of certain criteria defined in the guidelines (5, 6).

Percutaneous pulmonary valve implantation (PPVI) has revolutionized the treatment landscape as an alternative to surgery, and comparable results regarding the feasibility of valve implantation and the satisfaction of valve competency have been reported in patients with severe PR after TOF repair (7–9). On the other hand, RVOT is typically enlarged with various anatomies, and only a small number of patients with native RVOT are suitable for PPVI, especially when balloon expandible valves are to be used (10). The self-expandible new-generation pulmonary valve Pulsta THV® (Tae Woong Medical Co, South Korea) has been successfully used in patients with native RVOT and different anatomies (11, 12).

A key challenge is tailoring the treatment to each patient's unique anatomy and hemodynamic profile. Innovative approaches have been devised to create patient-specific pulmonary artery models and enhance preimplantation planning and decision-making (13). Recent advancements have enabled the development of patient-specific pulmonary artery models by customizing commercially available cardiovascular mock loops such as the ViVitro Super Pump (14). These mock loops, initially designed for general cardiovascular testing, were modified to replicate the physiological conditions of the pulmonary circulation. This study aimed to evaluate the feasibility and hemodynamic efficiency of Pulsta THV® patient-specific 3D-printed RVOT anatomy.

## Materials and methods

### Patients and valve implantations

Five patients who underwent Pulsta® THV between February 2020 and December 2021 were included in the study. Ethical approval was obtained from the Koç University Ethical Committee. The patients were diagnosed with severe pulmonary regurgitation due to transannular patch repair of TOF. All patients were evaluated using echocardiography and computerized tomography angiography (CTA). Magnetic resonance imaging was performed on asymptomatic patients. Indications for PPVI were defined according to previously described criteria (6). Percutaneous valve implantation was successfully performed in all cases. The device size was selected using pre-procedural images and the balloon-sizing technique as previously defined (15). After measuring the hemodynamic data of each patient's right ventricle and pulmonary arteries, injections were performed to visualize the RVOT and pulmonary artery anatomy. Angiographic measurements were performed from two angles at the largest moment of the cardiac cycle in systole. A sizing balloon (AGA Medical Inc., USA) was inflated in the RVOT, and a right ventricular injection was performed using a pigtail catheter inserted from the counter-side femoral vein. The balloon diameter blocking the contrast passage was measured and the Pulsta® THV size was selected to be 2 mm larger than this measurement.

### Patient-specific pulmonary artery 3d model generation

Computerized tomography (CT) scans were acquired using a multidetector CT scanner (Somatom Definition Flash) with 128 detector rows (Siemens Healthineers, Forchheim, Germany) with patients in the supine position. A prospective electrocardiography-gated multiphase scanning technique was used to reconstruct systolic phase images. CT scans were performed at 80 kV and 40–100 mAs with automatic exposure control. A non-ionic contrast medium (370 mg I/ml) at a 1.5 ml/kg contrast dose was administered intravenously, and the images were acquired using the bolus tracking method. Images were reconstructed with a slice thickness of 0.75 mm in the systolic phase interval of the cardiac cycle, in which the pulmonary artery had the widest diameter.

During image acquisition, artifacts due to breathing or the movement of the patients were avoided. Adequate signal density and contrast were obtained from 3D volumetric cardiovascular images of the RVOT, including the main pulmonary artery and branches.

**Figure 1:**
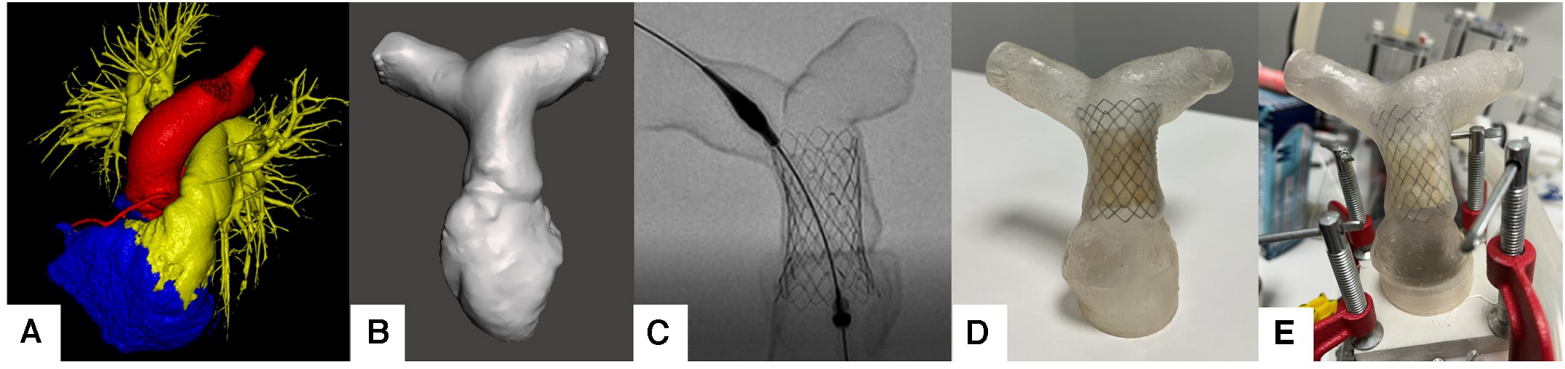
(**A**) Pulmonary artery segmentation of the patients. (**B**) STL model generation. (**C**) fluoroscopic evaluation of the valve placement. (**D**) Pulsta Valve 26 mm implantation on the 3D printed model. (**E**) Placement on ViVitro Pulse Duplicator system with in-house made mounts.

Then, 3D segmentation and modeling were performed on the patients’ CTA images. CTA images were segmented using image processing tools in “Inobitech DICOM Viewer Pro®” (Inobitech, Russia). The models mainly focused on the RVOT, the main pulmonary artery, and branches. The wall thickness was set to 1 mm. Digital Imaging and Communications in Medicine (DICOM) data were converted to the Standard Triangle Language (STL) form, and a segmentation process was performed (Figure 1A). After segmentation, hollow models were created by subtracting the segmented blood pool from the wall of the anatomical region (Figure 1B).

**Figure 2:**
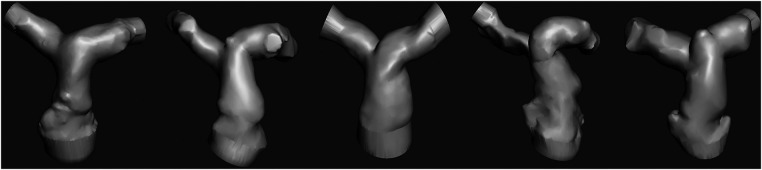
STL views of the patients.

Patient-specific pulmonary artery models for each patient are depicted in Figure 2. A Formlabs Form 3 (Formlabs Inc., USA) 3D printer was utilized, employing 80A resin, renowned for its biocompatibility and durability, to fabricate accurate replicas of the patients’ pulmonary arteries. The 80A resin was selected to closely mimic the mechanical properties of human vascular tissue. Valve placement was performed to mimic the real procedure under fluoroscopy (Figures 1C,D).

### Cardiovascular mock loop and hemodynamic measurements

**Figure 3:**
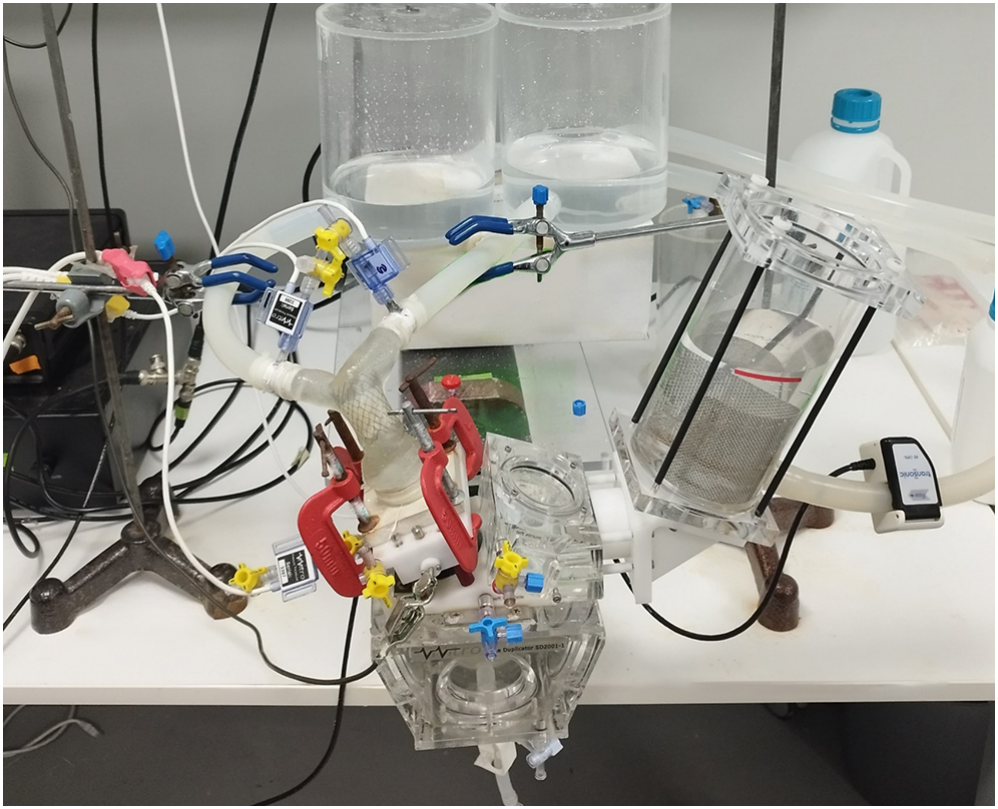
Pulsatile flow experiments. Measurement locations and 3D patient model connection ports are illustrated. Patient models were introduced in the mock-up single-ventricle circuit featuring patient-specific 3D printed mounts with Luer lock ports for pressure measurements. The pulse duplicator sets the mock-up system flow rate by adjusting stroke and stroke volume. Pulsatile pressure measurements were acquired from the MPA root and left/right PAs. The pulse duplicator's electromagnetic flowmeter (EM) was kept at its original place under the PA root. Another ultrasonic clamp on the flow meter was also used for the calibration of the EM flow meter. Fixtures were adjusted to avoid buckling of the patient models due to tensions on the silicone tubes.

In our study, we assessed the performance of the valves using a mock circulatory system designed to mimic the conditions of a single-ventricle pediatric patient as previously described in our earlier studies (16, 17). To ensure that the flow conditions closely resembled those in the patients, we modified the ViVitro pulse duplicator (Vivitro, Canada) with a 3D-printed fixture to test the patient-specific geometries (Figures 1E, 3).

During testing, the mock circulatory system was operated on with a systolic duration of 60% systole-to-diastole ratio of 0.6, maintaining a mean arterial pressure (MAP) of 20 mmHg. Distilled water was used as the test fluid at room temperature to ensure comparable hemodynamic conditions across different valve designs. The circulation system was tested at various stroke volumes to achieve a 3–5 L/MIN flow rate at 70 strokes per minute with a mean arterial model pressure of 20 mmHg (±0.5).

We measured and calculated the regurgitation fraction (RF) and pressure gradient. This was determined by measuring the ratio of forward and backward flows using an electromagnetic flowmeter available in the ViVitro system. We collected data from 10 pulses after the flow conditions reached a steady state. The Utah pressure transducers (Utah Medical Products, USA) were strategically placed in the ventricle and the right and left pulmonary arteries (Figure 3). The resulting data provided valuable insights into conduit selection by offering comparisons of RF and pressure gradients across a range of pediatric flow rates.

Five patient-specific pulmonary artery models, each representing a distinct systolic patient pulmonary artery geometry, were subjected to rigorous testing. Transcatheter pulmonary valves were assessed within these models, with attention to sizing, placement, and deployment. The angiographic measurements of the implanted and tested valve sizes are shown in Table 1.

**Table 1:** Anatomic measurements of the main pulmonary arteries (MPA) of patients were recorded by angiographic and balloon sizing methods. The patients underwent testing using prosthetic valve sizes both 2 mm larger and smaller than their implanted valve size. Nevertheless, it is of note that a 32 mm valve could not be accommodated within the 3D model of Patient 3, and Patient 4 was unable to be tested with a 34 mm valve, as 32 mm represents the upper limit of sizing in this case.

Patient number	Tested valve sizes (mm)	MPA dia. in Angio (mm)	Balloon dia. (mm)	Implanted size (mm)
Patient 1	26, 28, 30	25, 5	26, 7	30, 0
Patient 2	28, 30, 32	25, 5	28, 3	30, 0
Patient 3	28, 30	25, 9	27, 6	30, 0
Patient 4	30, 32	27, 0	30, 3	32, 0
Patient 5	28, 30, 32	25, 0	26, 4	30, 0

**Figure 4:**
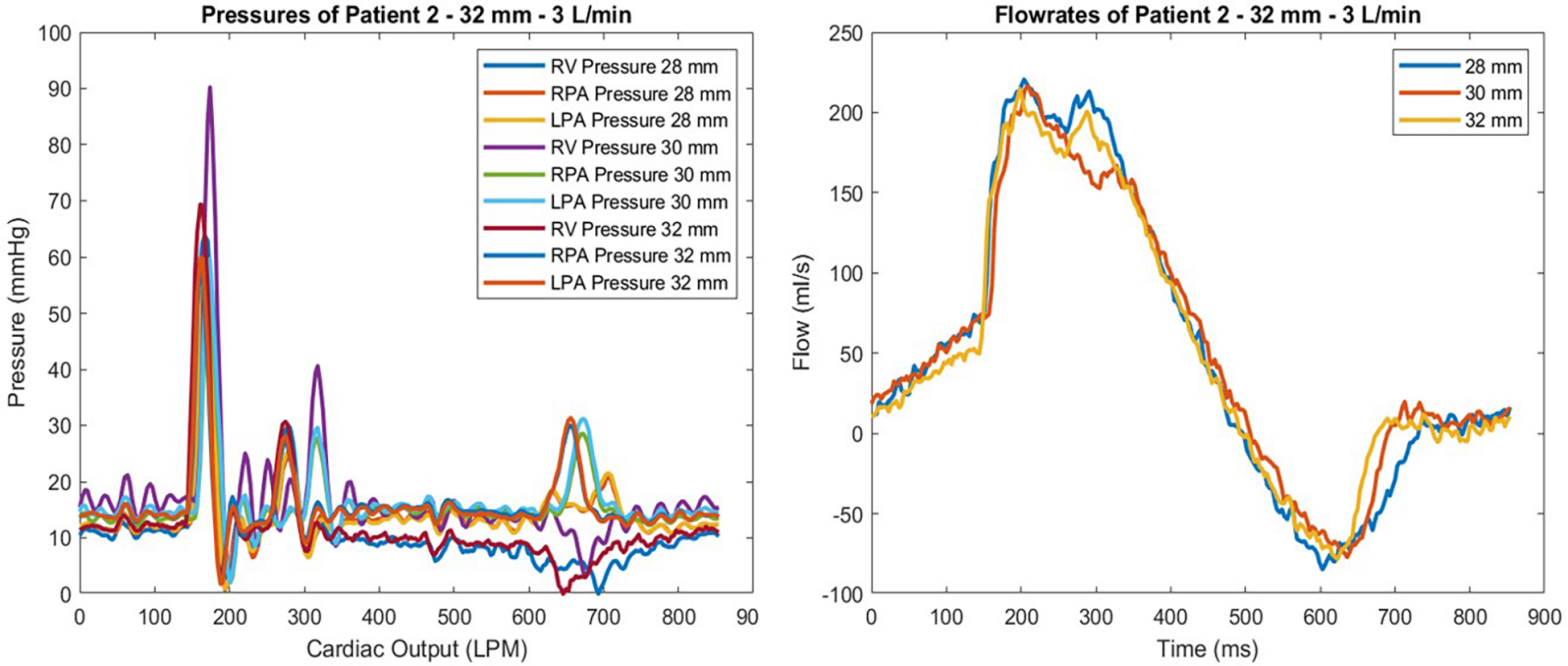
Pressure waveform of patient 2 at 3 L/min for all valve sizes tested are shown (left). Flowmeter measurements of the same test conditions are given (right). The highest gradient observed was with the 30 mm valve, which also showed the largest regurgitation by percentage.

Hemodynamic parameters, including regurgitation, pressure gradients, and flow rates, were calculated and analyzed during each testing iteration. measured the pressure gradients across transcatheter pulmonic valves (Figure 4).

## Results

A total of 39 experiments were conducted using five different patient geometries and several different valve sizes (26, 28, 30, and 32 mm) at 3, 4, and 5 L/min cardiac outputs at heart rates of 70 bpm and 60/40 systolic/diastolic ratios. All the data are summarized in Table 2.

**Table 2:** Summary of the findings for all patients and test conditions. Implanted valve sizes are underlined.

Patient #	Valve Size	CO (LPM)	Reg (%)	Std	Pg-L(mmHg)	Pg-R(mmHg)
Patient 1	26 mm	3	9, 93	1, 52	12, 13	7, 44
		4	11, 02	0, 38	13, 70	9, 64
		5	10, 76	0, 80	19, 23	19, 69
	28 mm	3	12, 89	1, 13	24, 94	24, 67
		4	12, 80	1, 29	27, 29	27, 01
		5	10, 26	0, 65	28, 68	29, 36
	30 mm	3	10, 02	0, 45	11, 76	11, 78
		4	10, 83	0, 60	17, 10	13, 89
		5	10, 43	1, 67	20, 31	17, 14
Patient 2	28 mm	3	18, 35	1, 00	7, 47	5, 94
		4	14, 40	1, 06	13, 16	10, 31
		5	13, 93	1, 15	17, 34	15, 63
	30 mm	3	14, 11	0, 81	26, 76	28, 42
		4	14, 55	0, 58	8, 92	13, 29
		5	15, 35	0, 66	14, 53	18, 93
	32 mm	3	12, 00	0, 76	11, 47	10, 03
		4	11, 36	0, 52	14, 85	13, 63
		5	13, 57	0, 74	17, 98	17, 85
Patient 3	28 mm	3	16, 46	2, 05	9, 16	7, 31
		4	15, 77	1, 72	14, 66	11, 11
		5	13, 99	0, 92	19, 26	14, 83
	30 mm	3	10, 84	0, 41	11, 13	8, 43
		4	11, 29	0, 69	13, 20	10, 23
		5	10, 33	1, 10	15, 61	13, 12
Patient 4	30 mm	3	18, 27	0, 30	9, 17	7, 16
		4	21, 39	2, 59	13, 72	9, 90
		5	23, 01	1, 31	19, 78	13, 10
	32 mm	3	14, 38	0, 47	10, 09	7, 72
		4	15, 60	0, 60	13, 35	9, 21
		5	16, 76	1, 68	16, 65	12, 89
Patient 5	28 mm	3	20, 86	1, 73	5, 99	1, 34
		4	19, 78	1, 37	10, 02	5, 32
		5	19, 39	1, 39	14, 42	9, 06
	30 mm	3	12, 35	1, 87	5, 92	3, 50
		4	11, 73	1, 01	7, 05	5, 24
		5	11, 86	0, 92	10, 18	9, 37
	32 mm	3	11, 21	0, 57	7, 16	2, 56
		4	10, 92	0, 52	9, 32	4, 46
		5	10, 78	1, 04	14, 28	9, 43

The minimum regurgitation for Patient 1 was achieved with a 26 mm valve at a 3 L/min flow rate of 9.9%. Maximum regurgitation occurred at 3 L/min and 4 L/min cardiac output (CO) with a 28 mm valve in that patient, which was almost 13%. The pressure gradients for these tests were also relatively higher than those of the other tests. The lowest pressure gradient occurred at the 26 mm valve with 3 L/min cardiac output (CO) as 12.13 mmHg from the left pulmonary artery (LPA) to the right ventricle (RV) and 7,4 mmHg from the right pulmonary artery (RPA). The 26 mm valve resulted in a superior gradient performance compared to the 30 mm with a 19.3 mmHg average gradient. The 28 mm valve showed the poorest performance for Patient 1, with the highest gradient and regurgitation values.

Patient 2 was tested using the 28, 30, and 32-mm valves. The 32 mm valve, which was the largest size, performed the best considering regurgitation and gradient performance. The lowest observed gradient was 11.35%, at a flow rate of 4 L/min. The resulting gradients were 14.85 on the left and 13.63 mmHg on the right branch. This patient geometry showed that a larger valve size performed better in our *in vitro* tests. However, the 28 and 30 mm valves performed similarly at 5 L/min CO; the 30 mm valve caused 15.35% regurgitation, while the 28 mm valve showed 13.9% regurgitation. The gradient values of these valve sizes were also compatible with a 28 mm valve left regurgitation of 17.3, and the right figure station was 15.63 mmHg. In contrast, the 30 mm valve left gradient was 14.52, and the right gradient was 18.9 mmHg (Figure 4).

Only two valve sizes, 28 and 30 mms, were tested for Patient 3. The 30 mm valve performed considerably better than the 28 mm valve at 5 L per minute. The 28 mm valve's regurgitation rate was 13.9%, whereas the regurgitation rate was 10.32% with the 30 mm valve. The gradient findings for the 30 mm valve were 15.60 on the left and 13.11 mmHg on the RPA. These values were much larger with the 28 mm valve, at 19.25 and 14.83 mmHg, respectively.

Similarly, Patient 4 was tested using two valve sizes: 30 mm and 32 mm. Maximum regurgitation occurred with the 30 mm valve with a 5 L/min cardiac output of 23.01%. However, a 32 mm valve resulted in 16.75% regurgitation. However, the pressure gradients were comparable. For the 30 mm valve, gradients were 19.78 on the LPA and 13.1 mmHg on the RPA, whereas they were 16.65 and 12.89 mmHg, respectively, for the 32 mm valve.

Patient 5 was tested using the 28, 30, and 32 mm valves. The 28 mm performed the poorest of all cardiac outputs, with a regurgitation value near 20%. Valve sizes of 30 mm and 32 mm were compared. The 32 mm valve performed slightly better. The regurgitation fractions were 11.85% for the 30 mm valve and 10.77% for the 32 mm valve at 5 L/min CO. Although regurgitation was considered higher with the 28 mm valve, the pressure gradient values were almost identical, approximately 14 mmHg on the left and 9 mmHg on the right.

## Discussion

Within the population of patients with TOF, clinical observations have revealed a diverse array of anatomies in the RVOT. These variations involve significant differences in vessel dilation, curvature radius, and RVOT angle as it extends from the right ventricle and the angle at the first pulmonary artery bifurcation (18). These anatomical variations make PPVI challenging for interventionalists. Additionally, anatomical variations profoundly impact the hemodynamic features and functions of valves (18).

Three-dimensional investigations prior to the procedure have recently become more popular for PPVI. However, these studies were related to the suitability of the RVOT anatomy for valve implantation (13). However, the complex flow characteristics of the RVOT after pulmonary valve implantation are unpredictable. Therefore, another *in vitro* experimental method is favorable for understanding the hemodynamic effects of PPVI.

3D cardiac modeling and printing are also becoming popular approaches for simulating complex congenital heart diseases (19, 20). The spatial relationship between the great arteries and ventricles can be shown more clearly, and according to data obtained from 3D modeling, surgical plans can be changed (21). For complex interventional procedures such as PPVI the benefit of the 3D models has also been reported. Jivanji et al. reported a self-expandible valve implantation procedure in the 3D heart model before the implementation procedure (22).

In our study, we investigated the hemodynamic characteristics of the new self-expandible valve Pulsta® THV in 3D models of patient-specific anatomy, using this novel modified Vivitro® pulse duplicator system. Different valve sizes were compared for different flow rates and cardiac output circumstances, with respect to pulmonary gradients and valve competency. The regurgitation fraction and pressure gradients of the pulmonary artery branches were measured and calculated, which were detected by the mock simulator, and showed consistency with the patients’ real angiographic and echocardiographic findings.

Implanted valve sizes showed regurgitation and pressure gradient performance similar to clinical data. Therefore, we concluded that the procedure described in this study could be a reliable pre-operation experimental tool. In addition, we observed that certain valves performed better than the clinical size in some of the experiments. For instance, regarding Patient 1, instead of the 30 mm valve that was implanted, a 26 mm valve size could have been implanted, which gave a better performance during tests. With a 26 mm valve, we obtained a lower regurgitation fraction and fewer pulmonary arterial gradients. This can be interpreted as the implantation of an unnecessary oversized valve during the procedure. This may be related to inadequate implantation experience at the beginning of the learning curve because of concerns regarding valve dislocation.

On the other hand, in Patient 2, although we implanted a 30 mm size valve, the test results showed that the best performance was obtained with a 32 mm valve. Thus, a 32 mm valve size selection could have been the best option for this patient if we had *in vitro* test results before implantation.

In addition, the best valve performance for Patient 3 was detected *in vitro* with a 30 mm valve, which was already implanted in the procedure. Hence, this was an *in vitro* validated result of the implanted valve size. In Patient 4, the implanted valve size also provided the best valve performance *in vitro*.

Regarding Patient 5, valves with sizes of 30 mm and 32 mm had similar *in vitro* test results, indicating that one of them could have been selected during the implantation procedure. This information gives a favorable advantage to the clinicians throughout the procedure in case of hesitation to avoid some complications related to oversizing.

The findings of this study also imply that smaller-diameter valves could be hemodynamically acceptable while avoiding the possible complications of oversizing, such as coronary compression. This study shows that this method may be used for decision-making processes by interventionalists for proper size selection in circumstances when it is challenging to evaluate which valve size should be used.

This study was designed as a proof-of-concept, covering five previously treated patients. An important advantage of evaluating valves before procedures using pulse duplicators is the reproducibility and confirmation of the results several times. This method can also be used prospectively in decision-making for PPVI procedures. Certain groups of patients, such as those with stenotic conduits, stenotic or hypoplastic pulmonary arteries, and/or bifurcation stenosis, are challenging to evaluate using our method. Further assessments of different implantation strategies, such as different positions of the valve or placement of valves at the pulmonary artery branches, in cases of infeasible RVOT anatomy, are also possible. Moreover, various stenotic conditions can be created or treated in 3D models, which can be replicated in our system for realistic *in vitro* evaluation. Although this study was conducted using only Pulsta THV ®, this system can also be used for valve selection by comparing different valve types or brands.

This study has some limitations. First, these models were created from the systolic images of the patients on CT angiography and did not completely reflect the whole anatomical change of the cardiac circle. The material we used (Formlabs 80 A Resin in 0.5 mm thickness) gave us similar mechanical test results with the previously reported pulmonary arterial tissue (23). However, the 3D model does not behave as the natural pulmonary arteries of the body since the surrounding tissue is excluded in this model. ECG-triggered full-cycle MRI images can provide a complete range of sizes of the pulmonic artery during the cardiac cycle, providing a more realistic assessment of the dimensions. Furthermore, this experimental mock model does not reflect the valves’ durability and long-term efficacy, which requires histological examination and measurements of the calcium deposit by using an accelerated mock pulse duplicator for a long duration. However, this was beyond the scope of this study.

Prior experiments with both blood and an aqueous glycerin solution damaged the experimental apparatus. Thus, a distilled water solution with the same density of blood was used instead in our experiments. Additionally, comparing the different fluids during testing showed no significant difference in the measurements (24, 25).

In conclusion, preprocedural investigation of valve function and hemodynamic characteristics with pulse duplicators is a promising tool for clinicians throughout the decision-making process, which is still open to complications. This research paper focuses on introducing the *in vitro* testing system of the PPVI in patient-specific anatomy 3D printed models. This is the first modification of the ViVitro pulse duplicator based on patient-specific anatomy. The use of Formlabs Form 3 3D printing technology with resin, electromagnetic flow meters, and custom-modified ViVitro Super Pump in conjunction with patient-specific pulmonary artery models has enabled a comprehensive assessment of percutaneous pulmonic valve performance. While our study primarily focuses on the Pulsta® valves, we believe the model's adaptability extends to various self-expanding valve platforms, and its utility can be applied within or in conjunction with existing core lab frameworks. This approach allows precise valve sizing, minimization of oversizing risks, and valuable insights into hemodynamic behavior before implantation. The data obtained from this experimental setup will contribute to advancing PPVI procedures and offer potential benefits in improving patient outcomes and safety.

## Data Availability

The original contributions presented in the study are included in the article/Supplementary Material, further inquiries can be directed to the corresponding author.
